# When Zoonotic Organisms Cross Over—Trueperella pyogenes Endocarditis Presenting as a Septic Embolic Stroke

**DOI:** 10.7759/cureus.7740

**Published:** 2020-04-20

**Authors:** Smit Deliwala, Thulasi Beere, Varun Samji, Philip J Mcdonald, Ghassan Bachuwa

**Affiliations:** 1 Internal Medicine, Hurley Medical Center, Michigan State University, Flint, USA

**Keywords:** trueperella, t. pyogenes, trueperella pyogenes, endocarditis, septic emboli, embolic stroke, embolic cva, zoonotic, actinomyces pyogenes, arcanobacterium pyogenes

## Abstract

Infective endocarditis (IE) remains a significant cause of morbidity and mortality worldwide, with numerous pathogens as culprits. We present a case of IE that evolved to a septic embolic stroke caused by an extremely rare bacteria Trueperella (T.) pyogenes that primarily infects non-humans. In contrast to most cases occurring outside the United States (US), this is the second case of T. pyogenes-associated endocarditis and the first to present as a stroke in the US. T. pyogenes has undergone numerous taxonomic revisions over the years since first being reported and characterized as Bacillus pyogenes in the 1800s. T. pyogenes is a zoonotic infection, and despite advancements in chemotaxonomic detection methods, Trueperella is often misidentified and under-diagnosed. Although epidemiological data is scarce, T. pyogenes infections have the propensity to cause endocarditis, and we aim to summarize all isolated reports of T. pyogenes infections that have been reported in the literature thus far.

## Introduction

The incidence of infective endocarditis (IE) is increasing in the United States and worldwide, caused by a broad spectrum of organisms [1]. Despite advancements in diagnostics and therapies for IE, the overall mortality rate has not changed over the past two decades, nor has the rate of in-hospital mortality [2]. In 1994, diagnostic criteria were developed to increase the detection of IE, known as the Duke criteria [3]. Strokes frequently complicate IE, and a temporal relationship between the two has been shown; cerebral septic embolic strokes are diagnosed with higher sensitivities by magnetic resonance imaging (MRI) than via clinical findings [4-5]. *Staphylococcus aureus* remains the most common cause of pyogenic endocarditis in the world, followed by viridans group Streptococci and Enterococci [6]. At the same time, 3% do not fit into the commonest of categories and 8% demonstrate negative blood cultures. Mitral and aortic valves remain the most affected sites at 41.1% and 37.6%, respectively, while heart failure 32.3%, non-stroke embolizations 22.6%, and embolic strokes 16.9% are the most common complications [2]. Embolic strokes originating from infected valves have a mortality rate nearing 30%, while the most common manifestation unilateral hemiparesis is seen in 45% of affected cases [7].

Over the years, T. pyogenes has undergone multiple revisions in its binomial name based on new chemotaxonomic data; this has paved the way for better identification systems and targeted therapy against the organism [8-9]. A thorough literature search confirmed our suspicions on the rarity of this organism, with a little under 50 reports of T. pyogenes infections in humans since its discovery over a century ago. We identified seven previously reported cases of T. pyogenes endocarditis, with only one similar case that was complicated by an embolic stroke confirmed on autopsy [10]. This case aims to bring into light the zoonotic organism T. pyogenes, provide a review of all the names it has been classified under, and guide future similar cases. T. pyogenes can pose a substantial health risk, with reports on endemic outbreaks in the past. The majority of the information drawn about T. pyogenes comes from veterinary data and studies, placing importance on isolated clinical cases [11].

## Case presentation

Over the years, a 52-year-old man developed a complicated medical history consisting of multiple admissions to our emergency department (ED). These were for alcohol intoxication interspersed with episodes of delirium tremens, multiple frostbite injuries requiring therapeutic amputations, chronic obstructive pulmonary disorder (COPD), hypertension, and chronic anemia. He was an active user of tobacco, alcohol, and cannabis products but never used injectable substances. He had a remote history of incarceration and was often displaced, seeking refuge in various shelters and group homes nearby. He was brought in by emergency medical services (EMS) after community residents noted alterations in behavior, with a last known normal time of earlier that morning. He was normotensive, tachycardic to 122, and febrile to 38.3 Celsius (101°F). He exhibited incoherent speech, inability to follow commands, drooling, dysphagia, dysesthesias in both feet, and right upper and lower extremity weakness with an overall National Institutes of Health Stroke Scale (NIHSS) score of nine. Blood cultures were drawn, and the patient was initiated on intravenous piperacillin-tazobactam 3.375 grams and vancomycin 1000 mg, with the concern for meningitis, alcohol withdrawal, or an acute stroke. He was intubated for airway protection while being maintained on a fraction of inspired oxygen (FiO2) of 30% and positive end-expiratory pressure (PEEP) of 5 mm/Hg while being sedated on dexmedetomidine and fentanyl infusions.

**Figure 1:**
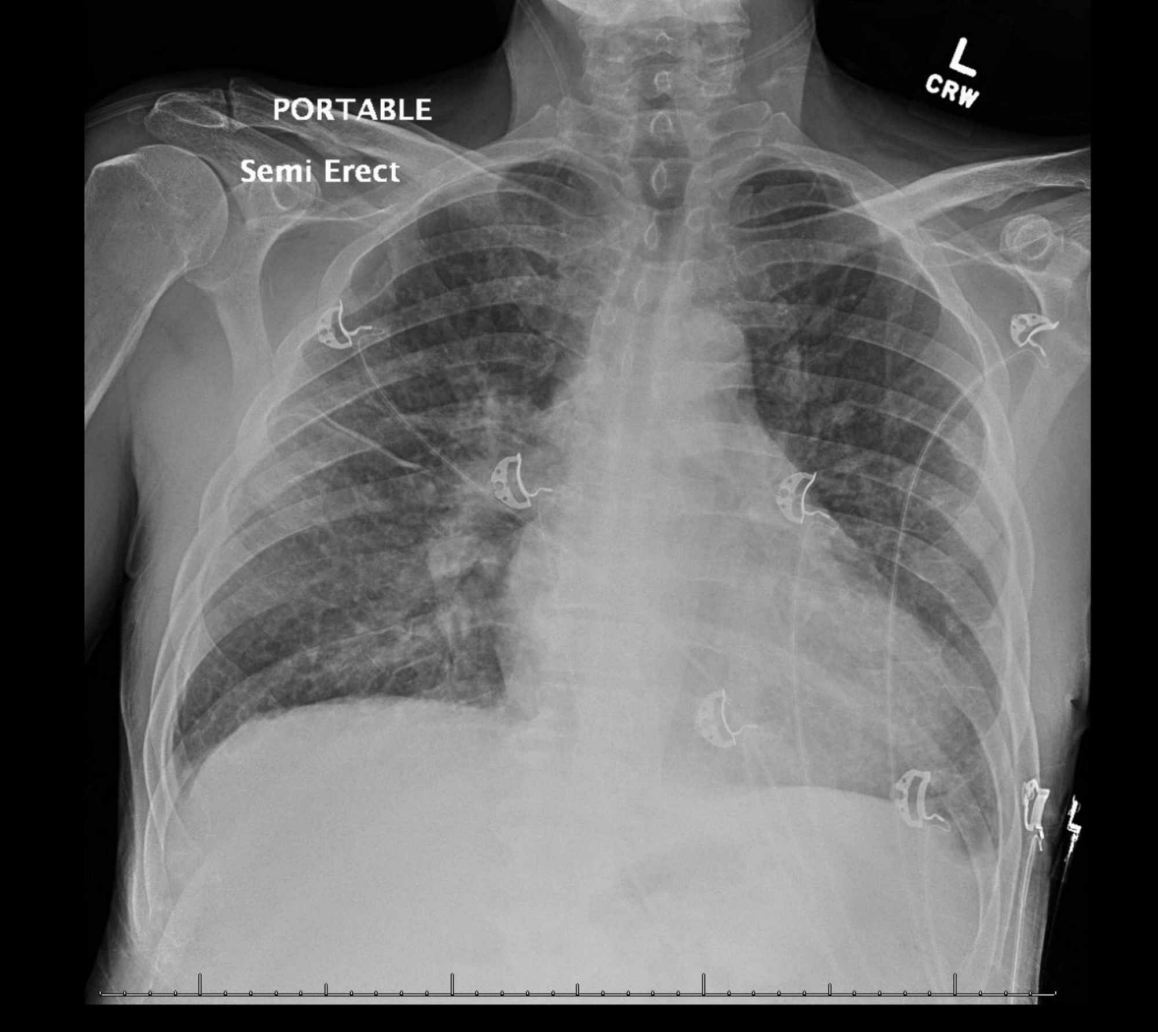
Chest radiograph on admission with mild hypervolemia

**Figure 2:**
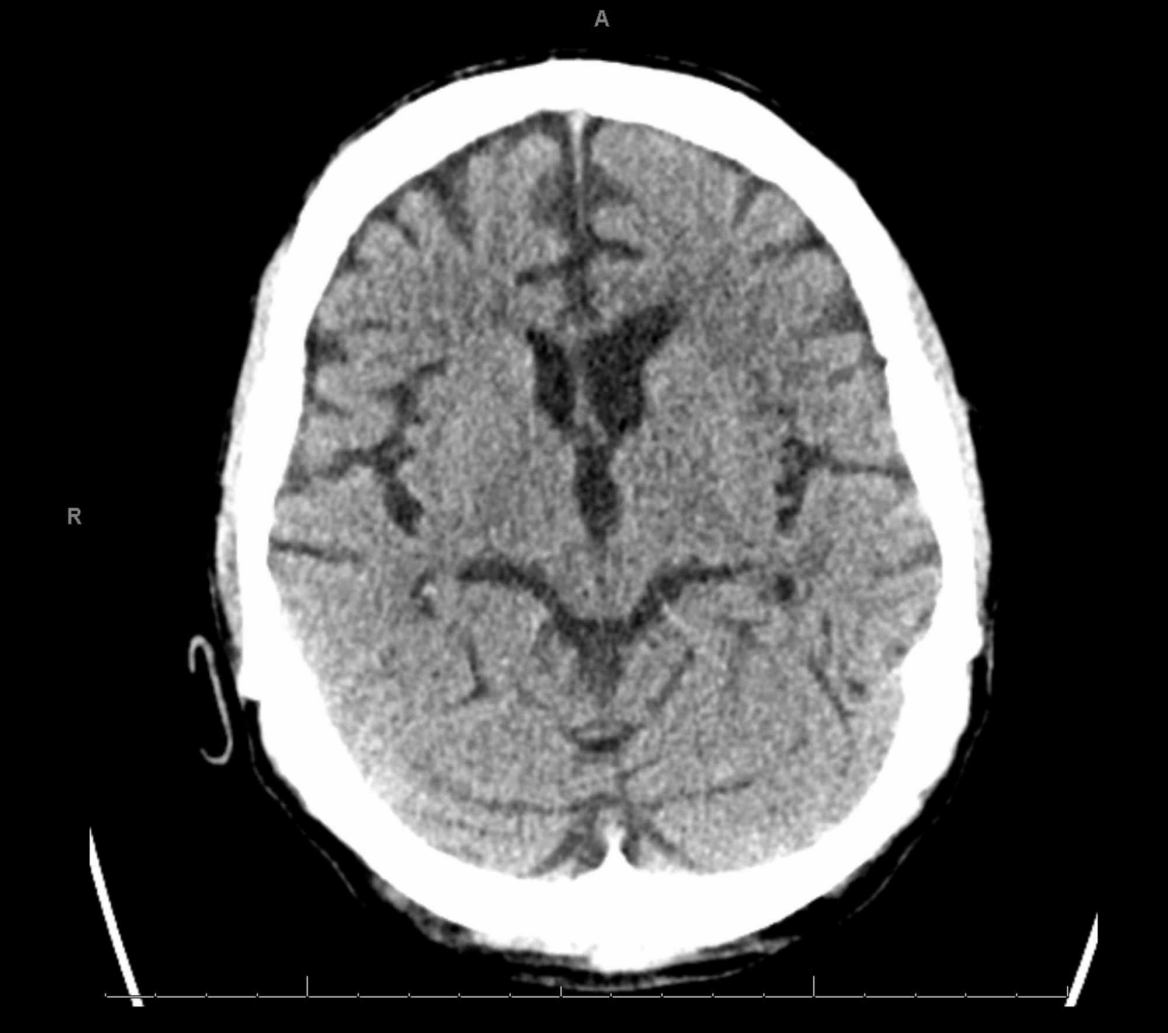
Computed tomography (CT) of the head without contrast on arrival

**Figure 3:**
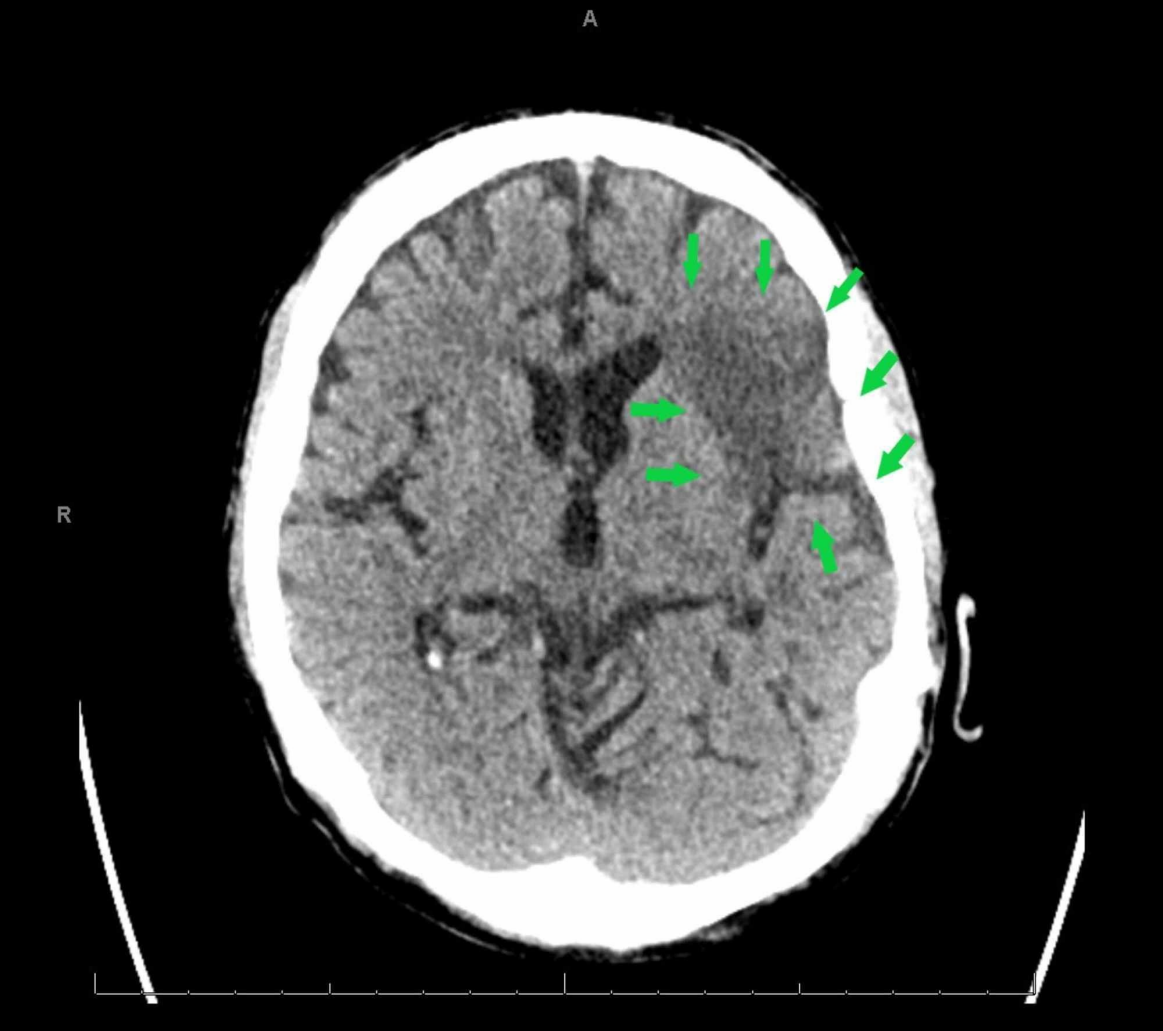
Computed tomography (CT) of the head without contrast revealing an acute left frontal infarct (arrows) 24 hours after admission

**Figure 4:**
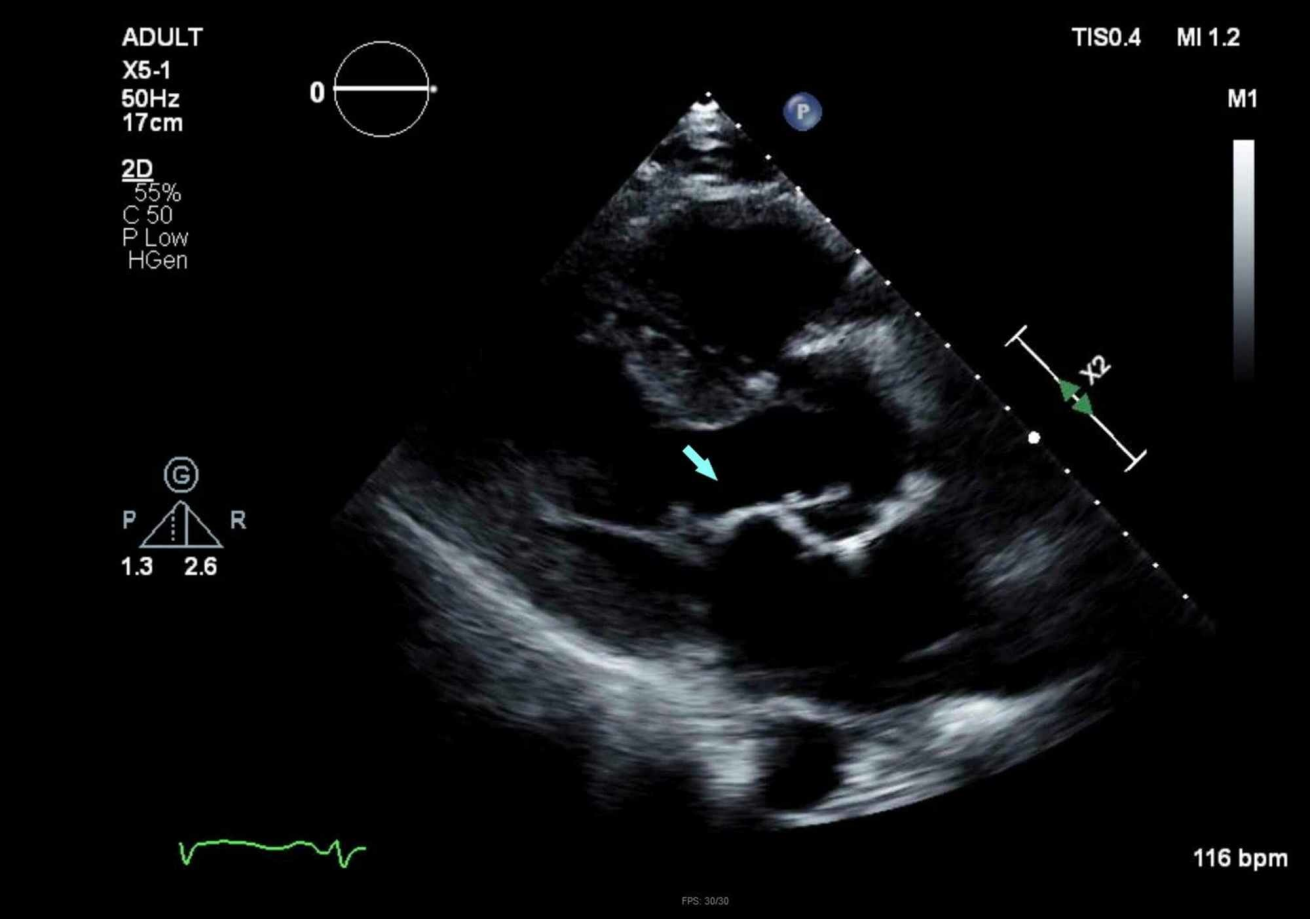
Transthoracic echocardiogram (TTE) revealing the mitral valve (arrow)

**Figure 5:**
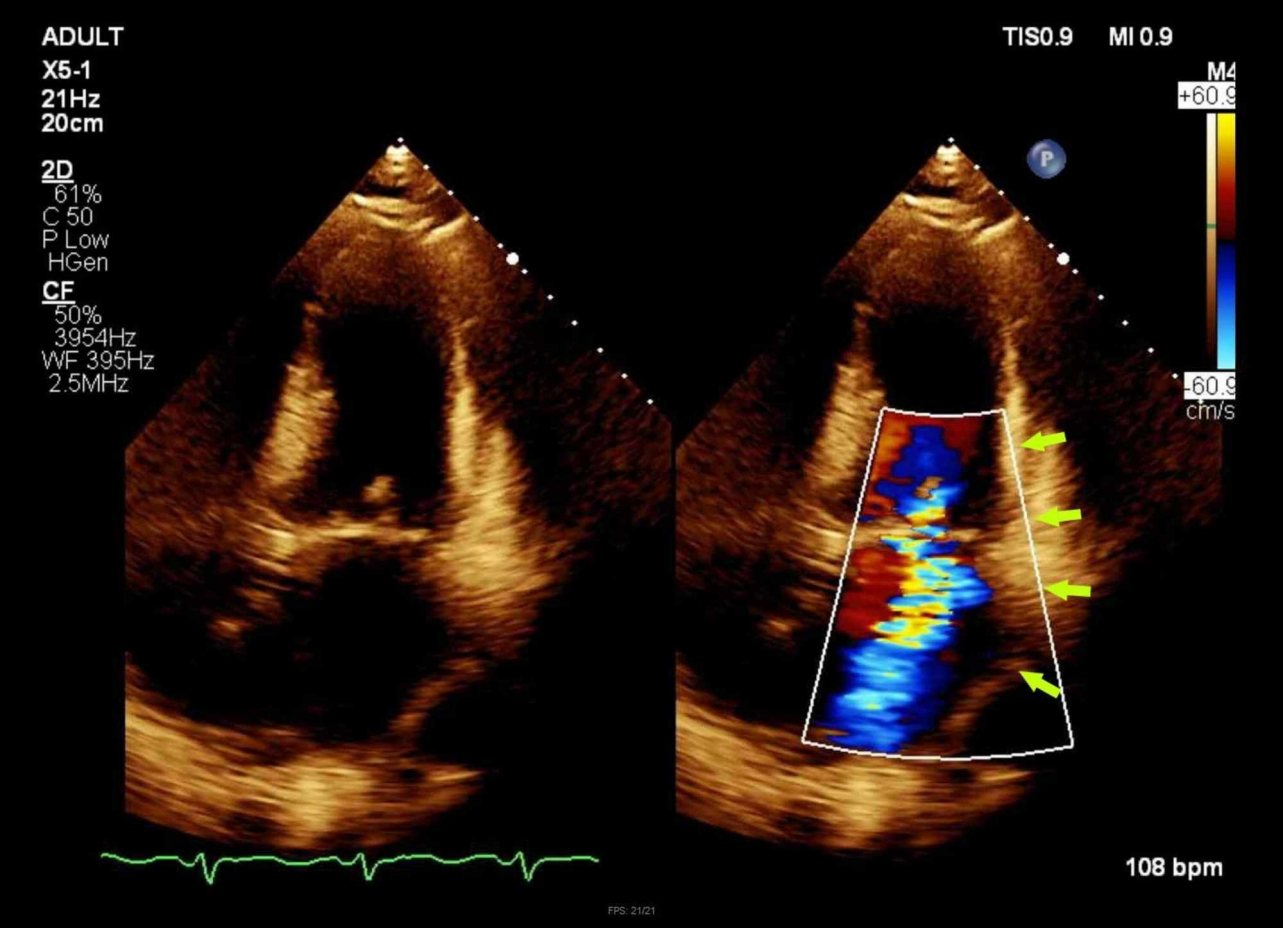
Transthoracic echocardiogram (TTE) revealing severe mitral regurgitation on Doppler flow (arrows)

**Figure 6:**
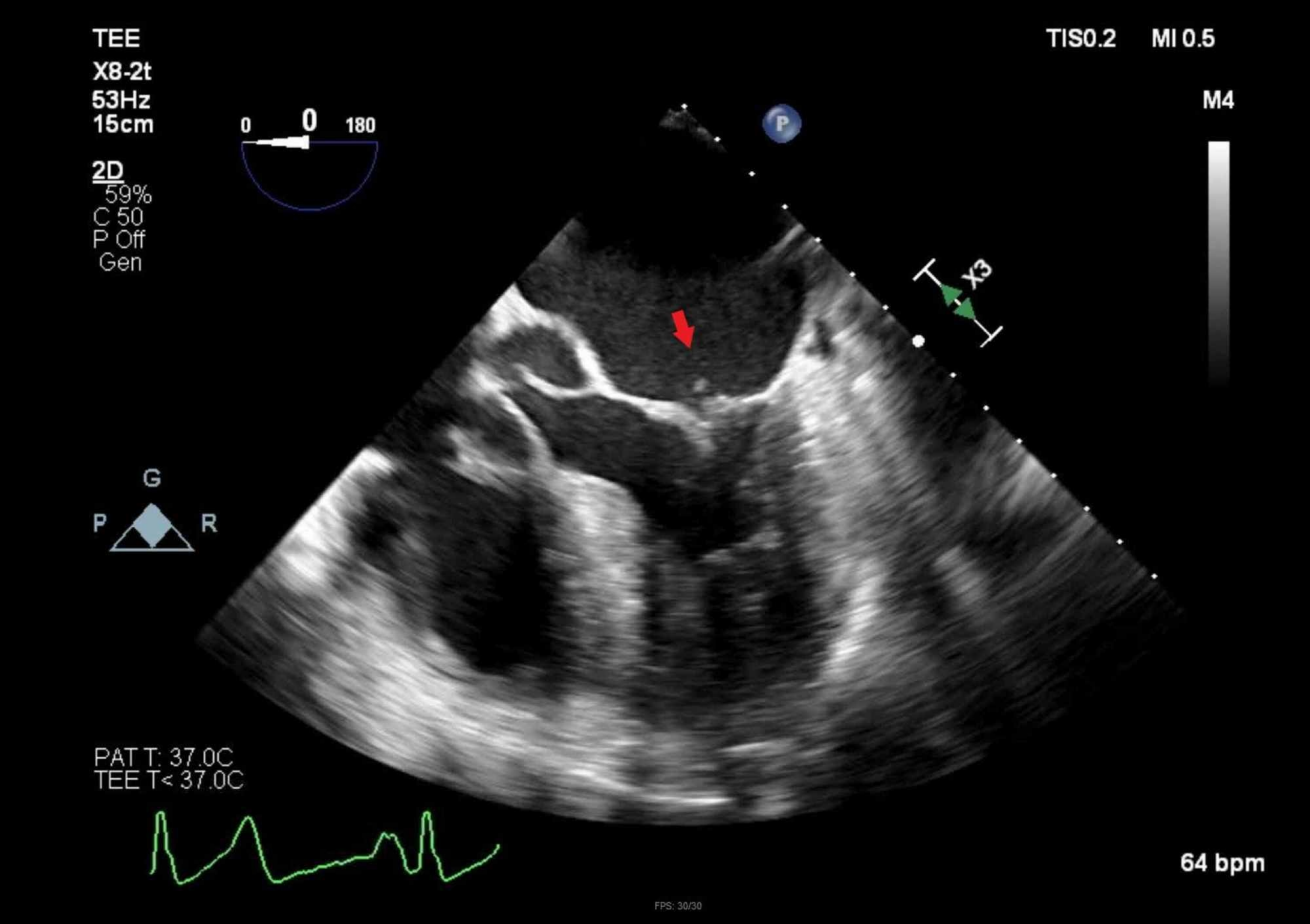
Transesophageal echocardiogram (TEE) revealing the mitral valve with vegetation (arrow)

**Figure 7:**
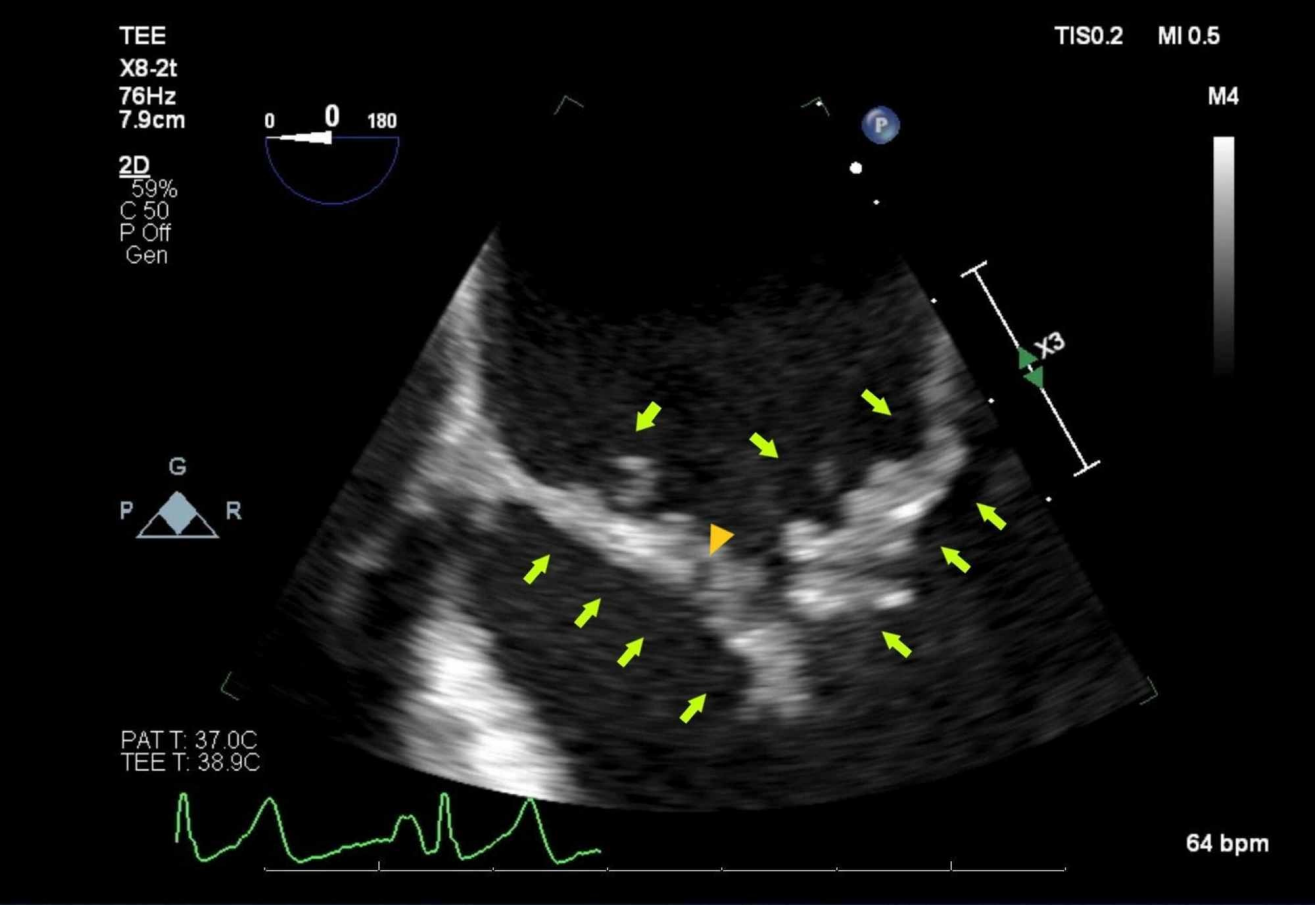
Transesophageal echocardiogram (TEE) revealing multiple vegetations along the mitral valve (arrow) and a central area of lucency consistent with an abscess (arrowhead)

A chest radiograph revealed evidence of hypervolemia (Figure 1). A non-contrast computed tomography (CT) scan of the head was unrevealing for any changes (Figure 2), and there was low suspicion for large vessel occlusion. A repeat CT scan due to the evolution of neurological signs was performed, demonstrating an acute left ischemic infarct (Figure 3). Laboratory examination on arrival was only remarkable for anemia with a hemoglobin of 9.1 g/dL, and a mild elevation in erythrocyte sedimentation rate (ESR) and C-reactive peptide (CRP) with both blood cultures bottles identifying gram-negative rods within 24 hours, with speciation to T. pyogenes 72 hours later (laboratory and imaging investigations in Table 1). Broad-spectrum antibiotic coverage was continued. Neurology consultation confirmed the presence of a stroke, prompting a workup for infective endocarditis considering his neurological sequela in the background of fever and positive cultures. A lumbar puncture was not pursued due to a lack of meningeal signs. A transthoracic echocardiogram (TTE) demonstrated a severely dilated left atrium with mitral regurgitation on Doppler (Figures 4-5); in contrast, a previous echocardiogram from his records revealed mild concentric left ventricular hypertrophy with a mild aortic root dilatation. A follow-up transesophageal echocardiogram (TEE) demonstrated foci on the mitral and aortic leaflets with an enlarged left atrium and an accompanying abscess (Figures 6-7). Despite blood cultures revealing T. pyogenes in both bottles, sensitivities were unavailable via our blood culture system, leaving us to resort to case reports to guide therapy. We switched his antibiotics based on these reports with the rationale of providing more targeted gram-negative coverage with intravenous ampicillin 2000 mg and gentamicin 360 mg. Atorvastatin and aspirin were added for secondary prevention, while intravenous furosemide was for hypervolemia. After 96 hours, his blood cultures were negative on three occasions. He developed transient transaminitis without clinical jaundice. Once extubated, he underwent conditioning via physical and speech therapy regularly. He was safely transferred to a tertiary center with advanced cardiac care for surgical valve candidacy due to the level of vegetation and abscess on echocardiogram. At the tertiary facility, the patient was ineligible for surgery due to overall morbidities and debilitated status, and the infectious disease team recommended to continue intravenous penicillin G for 12 weeks and then maintain on doxycycline for one year with periodic check-ups.

**Table 1:** Investigations during infection workup HIV: human immunodeficiency virus; CRP: C-reactive protein; ESR: Erythrocyte sedimentation rate; AST: aspartate aminotransferase; ALT: alanine aminotransferase; CT: computed tomography; TTE: transthoracic echocardiogram

Investigation	Result
Sodium on arrival	136 MEQ/L
Potassium on arrival	4.2 MEQ/L
Creatinine on arrival	0.7 MG/DL
Anion gap	7 MEQ/L
White blood cell count on arrival	7.5 K/UL
Hemoglobin on arrival	9.1 G/DL
Platelets on arrival	166 K/UL
HIV Ag/Ab 4^th^ generation screen	Negative
Urinalysis	Positive for blood
Ethanol	Negative
Toxicology screen	Negative for commonly abused substances
Blood culture x 2 on arrival	Positive for Trueperella pyogenes
Ammonia	35 UG/DL
Brain natriuretic peptide on arrival	513.2 PG/ML
Brain natriuretic peptide after 72 hours	3817.5 PG/ML
Lactate after 72 hours	1.2
Blood culture x 2 after 96 hours	Negative
CRP	24.20 MG/L
ESR	58 MM/HR
AST on arrival	18 U/L
ALT on arrival	< 9 U/L
AST in 96 hours	529 U/L
ALT in 96 hours	455 U/L
AST on discharge	44 U/L
ALT on discharge	194 U/L
CT of the head without contrast on arrival	No acute intracranial hemorrhage, mass effect or midline shift noted. Changes are due to chronic small vessel ischemic changes with diffuse cerebral atrophy (Figure 2)
CT of the head without contrast 24 hours after arrival	Interval development of a relatively large region of abnormally decreased attenuation in the posterior left frontal region consistent with an acute infarct (Figure 3)
TTE	Ejection fraction >70%. Left ventricle normal size with severe dilatation of the left atrium with a volume index of 53 ml/m^2 ^(Figures 4 & 5)
TEE	severe mitral regurgitation with clear vegetations on both leaflets on the atrial side of the mitral valve and a thickened anterior mitral valve leaflet with significantly sized vegetations without clear lucency, complemented with small vegetations on the ventricular side of the aortic valve with mild-to-moderate aortic insufficiency (Figures 6 & 7)
Modified barium swallow	moderate/severe oral dysphagia and moderate pharyngeal dysphagia

## Discussion

**Figure 8:**
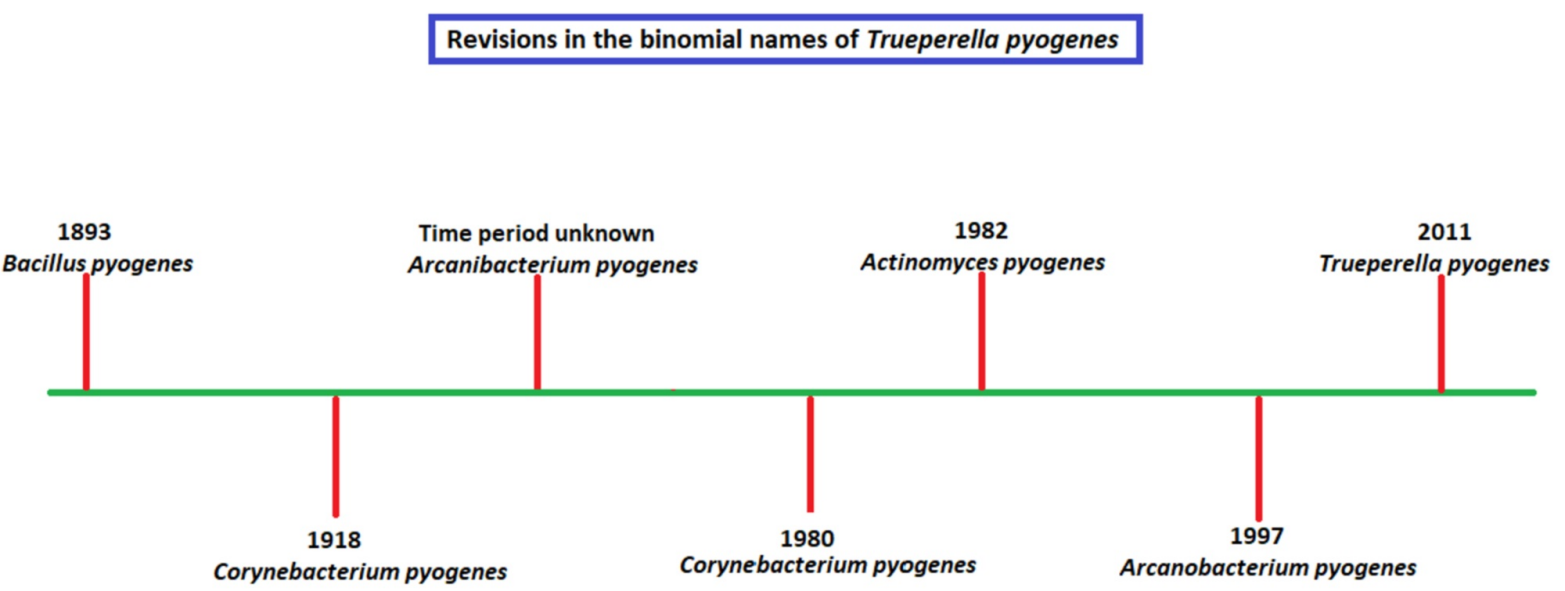
Various taxonomic revisions of Trueperella pyogenes since inception

T. pyogenes was first described as Bacillus pyogenes in 1983, an infection almost exclusively seen in animals. It is known to frequently colonize the mucous membranes of the body of many domestic animals and is primarily an animal pathogen causing pyogenic infections [12-13]. It is a gram-positive, pleomorphic, non-spore-forming, non-motile, non-capsulated, facultative anaerobic rod, characterized by a zone of beta-hemolysis on blood agar with catalase negativity [11]. Figure 8 encompasses all the taxonomic revisions of T. pyogenes over the years. Historically, it wasn't straightforward to differentiate between Actinomyces species, as most clinical laboratories were not equipped to detect them and they were often identified as Streptococci. This mismatch was due to their aerobic properties, gram-positive reaction, beta-hemolytic architecture on blood agar, and its subsequent reaction with Lancefield group G [14-15].

T. pyogenes contains an armory of virulence mechanisms, such as hemolysin, pyolysin, and cytotoxic abilities, toward immune cells like macrophages. At the same time, various external adherence mechanisms and fimbriae contribute to its pathogenicity [12]. They cause a variety of infections, ranging from liver abscesses to pneumonia, mastitis, foot infections, endometriosis, septic arthritis, and cutaneous eruptions in cattle species [15]. T. pyogenes has not been isolated as part of the healthy human biome and is pathologic by its very nature, with a scarcity of reported cases in humans [12,14,16].

T. pyogenes endocarditis has been reported in various parts of the world. Despite a pre-disposition to tropical regions, it has been isolated in Canada, Spain, China, and the United States [17]. Our patient demonstrated risk factors to develop native valve endocarditis, including advanced age, poor dentition, homelessness, and possible exposure to zoonotic organisms. Although T. pyogenes infections in humans are infrequent, when they do occur, they can have devastating consequences, as evidenced by their slew of virulent mechanisms [2]. It often leads to infections such as meningitis, septic arthritis, abdominal infections, lumbar abscesses, and thrombocytopenic purpura [17]. Human-to-human transmission has never been estimated, although the possibility of an endemic spread was demonstrated from a case series of 10 reports with frequent outbreaks of T. pyogenes leg ulcers in Thailand, alluding to its virulence and the possibility of it being a skin contaminant [16,18]. In our patient, a prior history of occupational exposure or the possibility of being near a farm or agricultural site during his episodes of homelessness, with the ability to acquire a skin contaminant, are possible risk factors for developing a significant T. pyogenes infection. Another likely explanation to develop the overwhelming infection could be the immunosuppression exhibited by our patient based on his severe nutritional status and overall debilitation, with multiple co-morbidities and a poor social fabric. Data on antimicrobial guidance is rare in humans, with the majority coming from veterinary studies. Beta-lactams, macrolides, lincosamides, and tetracyclines are the antibiotics of choice. However, there has been a recent growing concern for T. pyogenes resistance due to the robust use of these antibiotics in agriculture [9,19]. Typically, complete eradication of microorganisms requires prolonged therapy due to deep-seated bacterial densities within the valve.

## Conclusions

Trueperella pyogenes was first discovered as Bacillus pyogenes in the 1800s and has undergone various taxonomic revisions since then. It is a zoonotic organism that frequently infects cattle. Although its presentation is incredibly rare, when it does infect humans, it seems to have a propensity to cause endocarditis. The absence of animal contact does not preclude the ability to acquire the infection. T. pyogenes is often seen embedded in mixed infections or characterized as Streptococci so an isolated case of Trueperella should be concerning. T. pyogenes has numerous virulence and evolutionary mechanisms that cause serious infections in humans although more research is required to ascertain their pathogenicity. Resistance to antibiotics is an emerging problem, with resistance noted to macrolides, lincosamides, and recent reports of low activity against tetracyclines due to the robust use of antibiotics in agriculture, although most infections in humans seem to be susceptible to penicillins, macrolides, aminoglycosides, and cephalosporins.
